# Different Approaches to the Treatment of Radicular and Related Cysts Associated with Nasal Mucosa in the Maxilla: A Case Series

**DOI:** 10.3390/jcm15062411

**Published:** 2026-03-21

**Authors:** Ömer Uranbey, Kamil Nelke, Furkan Diri, Burcu Gürsoytrak, Füruzan Kaçar Döger, Lale Okumuş, Agata Małyszek, Maciej Janeczek, Filip Kulewicz, Maciej Dobrzyński

**Affiliations:** 1 Department of Oral and Maxillofacial Surgery, Faculty of Dentistry, Aydın Adnan Menderes University, Aydın 09100, Turkey; 2 Maxillo-Facial Surgery Ward, EMC Hospital, Pilczycka 144 Street, 54-144 Wrocław, Poland; 3 Department of Medical Pathology, Faculty of Medicine, Aydın Adnan Menderes University, Aydın 09100, Turkey; 4 Department of Biostructure and Animal Physiology, Wrocław University of Environmental and Life Sciences, Cypriana K. Norwida 31 Street, 50-375 Wrocław, Poland; 5 Aesthetic Medicine and Dental Implantology Specialized Practice, Śniadeckich 53, 51-604 Wrocław, Poland; 6 Department of Pediatric Dentistry and Preclinical Dentistry, Wrocław Medical University, Krakowska 26 Street, 50-425 Wrocław, Poland

**Keywords:** maxillary bones, radicular cyst, nasal mucosa, treatment options, bone regeneration

## Abstract

Radicular cysts (RCs) represent the most frequent inflammatory cystic lesions of the jaw, typically arising from non-vital teeth. While standard management via enucleation is well-documented, complex cases involving the anterior maxilla present significant surgical challenges due to their proximity to the nasal cavity floor (NCF) and the maxillary sinus floor (MSF). This report provides a comprehensive revision of a clinical case series involving seven patients (ages 17–50) treated with multimodal surgical and regenerative protocols. The patients were stratified into five distinct anatomical risk groups (A–E) based on the integrity of the bony boundaries and the presence of oronasal communications. The treatment strategies combined meticulous cyst enucleation with advanced regenerative techniques, including platelet-rich fibrin (PRF), allogeneic and xenograft bone substitutes, and local flaps such as the buccal fat pad (BFP). The results across all seven cases demonstrated favorable clinical and radiographic outcomes, with no instances of oronasal fistula formation or recurrence during follow-up periods ranging from 12 months to three years. This report emphasizes the necessity of structured anatomical stratification and multimodal planning to ensure scientific precision and surgical predictability in the management of complex maxillary lesions. The differences between approaches towards the nasal cavity and maxillary sinus have to be highlighted. Further studies with larger cohorts are warranted to evaluate the long-term outcomes of different treatment modalities.

## 1. Introduction

The extension of various cysts into the nasal cavity and/or maxillary sinuses represents a significant surgical challenge. To restore adequate bone volume, shape, and position and to maintain tooth stability, various surgical approaches may be employed. Cysts in the jawbone can be classified as inflammatory, neoplastic, and developmental, according to the World Health Organization [1]. The RC is an inflammatory lesion believed to originate from the proliferation of epithelial cell rests of Malassez in the apical region of a tooth with an infected pulp. Traditional radiographic imaging and cone beam computed tomography (CBCT) are insufficient for a definitive diagnosis of RCs; histological examination of biopsy specimens remains essential [2]. RCs typically appear on conventional radiographs and CBCT as osteolytic lesions at the periapical region of a tooth with an infected, necrotic pulp. A cystic lesion is typically suspected when the lesion measures more than 2 cm in diameter [3,4]. A cyst should be considered when the lesion exhibits a thin corticated border [5,6]. The majority of RCs are asymptomatic. Typically, patients present with complaints of slow-growing swelling. Initially, the growth is osseous and stiff, but as the cyst enlarges, the bone becomes thinner despite subperiosteal bone deposition. Fluctuation becomes evident when the cyst fully elevates the overlying bone. In the maxilla, swelling typically presents buccally and palatally, whereas in the mandible, it is primarily buccal and only rarely lingual. The fundamental requirement for an RC is the existence of a tooth with a devitalized pulp [7]. Pain and infection may accompany these lesions; however, they are generally asymptomatic unless secondarily infected. However, in some patients, pain persists despite the absence of clinical evidence of infection and histologic evidence of acute inflammation following cyst removal. Conversely, in some patients, there was no clinical evidence of pain despite clinical signs of infection [8,9,10]. RCs are the most common cysts of the jaw and account for approximately half of all odontogenic cysts [11]. Large-scale retrospective studies have reported that these lesions comprise 50–60% of all odontogenic cysts, with a slight male predominance; reported male-to-female ratios range between 1.3:1 and 1.5:1 across diverse populations [11,12]. The prevalence of these conditions is highest in the third decade of life. The prevalence of this condition is higher in men than in women [7]. The anterior maxillary and mandibular premolar regions are the areas where radicular cysts are more common than other areas of the jaw [13]. In contrast to developmental and neoplastic cysts, which are self-perpetuating and often require surgical removal, inflammatory lesions may resolve following non-surgical root canal treatment [2,14,15,16]. In instances where the healing process following root canal treatment is unsuccessful, surgical intervention should be considered as a treatment option. It is recommended that decompression therapy be administered prior to enucleation in cases of close proximity to vital structures, such as the maxillary sinus, mental foramen, and mandibular canal [17,18]. However, complete enucleation is known to provide total removal of the cyst and faster healing of the cavity [19]. In the case of large RCs, platelet-rich fibrin (PRF), a concentrate of growth factors that stimulate healing of the bone cavity, can be used [20]. More recently, PRF has been shown in systematic reviews to promote angiogenesis, osteoblastic differentiation, and immunomodulation in alveolar bone defects, and to serve as a scaffold in combination with bone substitutes [21,22]. The clinical benefits of platelet-derived biomaterials have also been supported by recent evidence showing that concentrated growth factors and injectable PRF significantly reduce postoperative complications and enhance soft-tissue recovery in oral surgery [23]. In addition, in maxillary cases, buccal fat pad flaps can be used to reconstruct the bone defect with low morbidity and few complications [24]. In the presence of advanced mobility in the teeth located in the enucleation area, a stable tissue is created, and bone healing is supported by splinting the teeth together after endodontic apicoectomy procedures [25]. In some cases, RCs may recur due to persistent or recurrent infection in the root canals of the retained teeth [2,26]. Table 1 presents the differences and similarities between the surgery in the floor of the nasal cavity and the dental recess of the maxillary sinuses.

Based on Table 1, we stratified lesions according to the integrity of the bony boundaries and the presence (or risk) of communication with the NCF and/or MSF. The following lesions were considered: (i) contained (Group A), (ii) adjacent with thin residual bone (Group B), (iii) communicating with a single compartment (Group C), (iv) compromising both NCF and MSF with shared wall loss (Group D), or (v) occupying both cavities (Group E). This anatomy-driven stratification directly guided treatment selection, ranging from single-stage enucleation with regenerative filling (Groups A–B) to two-layer mucosal separation and barrier-based closure when communication was present (Group C), and to staged decompression/marsupialization followed by definitive enucleation when combined cavity involvement or unstable compartmental separation was anticipated (Groups D–E). Exceptionally, in edentulous patients with excessively large cystic defects, especially in severely atrophic maxillae where compartmental separation cannot be predictably achieved, salvage creation of a controlled communication between the cyst cavity and the maxillary sinus may be considered.

Possible cysts and tumors that can be found in the anterior maxillary area might include radicular and residual cysts, dentigerous cysts, nasopalatine duct cysts, odontogenic keratocysts, and benign odontogenic tumors such as ameloblastoma [27]. Among these entities, RCs represent the most frequent odontogenic cysts, accounting for approximately 50–60% of cases reported in large epidemiologic series [28]. However, data remain limited regarding optimal surgical and regenerative strategies for RCs of the anterior maxilla when the lesions involve the nasal mucosa or maxillary sinus membrane, prompting the need for the present case series. Different treatment approaches were applied to RCs extending to the nasal cavity floor, spreading towards the maxillary sinus, and/or spreading from the maxillary bone process towards the nasal cavity, and their clinical and surgical outcomes are presented and discussed. The aim of this study was to present a structured descriptive case series of anterior maxillary cystic lesions involving or threatening the nasal cavity floor and/or maxillary sinus floor, and to illustrate how an anatomy-based stratification may guide surgical decision-making.

## 2. Case Series Design and Case Descriptions

This study was designed as a retrospective descriptive case series of seven surgically managed anterior maxillary cystic lesions with radiologic proximity to or involvement of the nasal cavity floor (NCF) and/or maxillary sinus floor (MSF). The present series was not intended as a consecutive epidemiologic cohort; rather, cases were purposively selected to illustrate different anatomy-based surgical scenarios and management strategies encountered in this region.

Inclusion criteria were: (i) the presence of an anterior maxillary cystic lesion associated with the NCF and/or MSF on CBCT; (ii) surgical treatment performed with adequate preoperative, intraoperative, and postoperative documentation; (iii) histopathologic confirmation of an odontogenic cystic lesion; and (iv) availability of follow-up data. Cases lacking histopathologic confirmation or sufficient imaging and follow-up documentation were not included.

Treatment planning was based on clinical examination, tooth vitality and endodontic status, CBCT assessment of lesion extent, integrity of residual bony boundaries, and the presence or anticipated risk of communication with the NCF and/or MSF. Before surgery, each case was assigned to an anatomic risk group (A–E) according to Table 1. This anatomy-driven grouping was used as a practical therapeutic framework rather than a validated scoring system. In general, lesions with preserved compartmental separation were managed with single-stage enucleation with adjunctive regenerative support when needed, whereas lesions with established communication, shared wall loss, or uncertain closure required barrier-based separation, additional soft-tissue support, grafting, or staged surgical treatment. Additional decisions such as endodontic treatment, apicoectomy, tooth extraction, splinting, and choice of grafting material were individualized according to tooth prognosis, defect size, mucosal status, and future prosthetic or implant-related needs.

This case series selected illustrative clinical situations to demonstrate the range of surgical approaches that may be required in anterior maxillary cystic lesions involving adjacent sinonasal structures.

The key clinical and surgical data for all patients are summarized in Table 2. While each case presented a unique anatomical challenge ranging from incidental findings to extensive destruction of the nasal floor, all seven cases followed the anatomy-driven stratification protocol (Groups A–E) to guide the selection of regenerative materials and surgical access.

### 2.1. Case One

**Figure 1:**
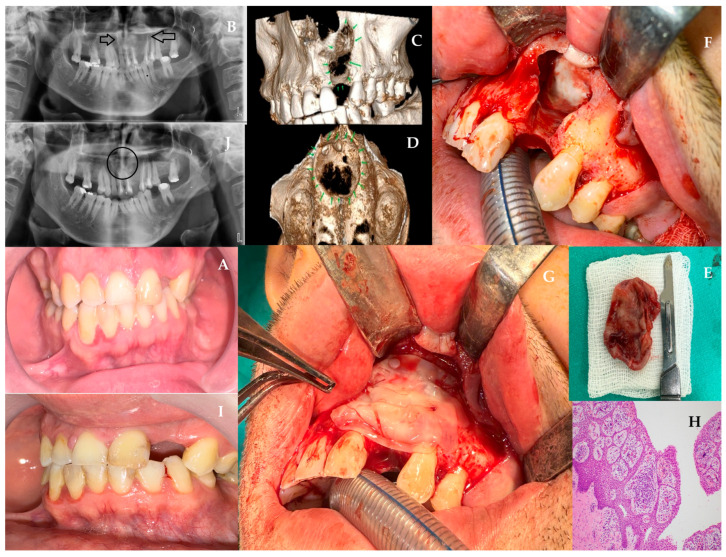
(**A**) Intraoral view demonstrating missing tooth 22 and intact mucosa. (**B**) Panoramic radiograph showing a well-defined unilocular radiolucency extending between teeth 11–25 (black arrows). (**C**,**D**) CBCT reconstructions revealing corticated borders and nasal floor involvement. (**E**) Intraoperative view of cyst enucleation (**F**)—Placement of absorbable collagen sponge (BIOPAD) over perforated nasal mucosa. (**G**) Layered placement of L-PRF membranes within the cyst cavity. (**H**) Histopathology showing non-keratinized stratified squamous epithelium with chronic inflammatory infiltrate. (**I**,**J**) 12-month postoperative clinical and radiographic views demonstrating complete healing without recurrence (black circle).

A 33-year-old male patient was admitted to our clinic due to the identification of a radiolucency on radiographic imaging during routine examinations. The patient had no history of systemic disease and reported no use of tobacco or alcohol. At the time of initial presentation to our clinic, there were no signs of extraoral swelling. On palpation, the area was firm and non-fluctuant. Intraoral examination revealed that tooth number 22 was missing (Figure 1A). The patient reported a history of trauma to the area, stating that tooth number 22 had initially undergone root canal treatment but was later extracted at an external clinic due to persistent symptoms. Panoramic radiography (Figure 1B) and CBCT (Figure 1C,D) imaging revealed a well-defined, unilocular, corticated radiolucency extending from tooth number 11 to tooth number 25. Aspiration of the lesion using a 2.5 cc syringe yielded cystic fluid. Based on these clinical and radiographic findings, the lesion was diagnosed as a residual type of RC, likely resulting from incomplete enucleation of a RC originally associated with tooth number 22. The vitality of associated teeth was evaluated before surgery, and root canal treatment was planned for the devitalized teeth 11, 21, 23, 24. The patient was scheduled for surgery under general anesthesia after completion of the endodontic treatment. The full-thickness mucoperiosteal flap was elevated through an intraoral incision extending from the distal of tooth 11 to 24, supported by vertical releases. After identifying the bony perforation, the remaining thin cortex was removed. Subsequently, intraoral cyst enucleation was performed (Figure 1E). An absorbable sponge barrier (BIOPAD, Euroresearch, Milan, Italy) was applied to the base of the perforated nasal mucosa and roof of the cyst cavity (Figure 1F). This barrier has been shown to promote granulation tissue formation and regeneration, thereby preventing nasal mucosa collapse into the cyst cavity. L-PRF was prepared according to the original protocol of Dohan Ehrenfest et al. [29] Venous blood was drawn into 9-mL glass-coated tubes without anticoagulant and immediately centrifuged at 400 *g* (2700 rpm) for 12 min. The fibrin clot formed between the plasma and red cell layers was gently retrieved, the erythrocyte portion was trimmed, and the clot was compressed in a sterile PRF box to obtain 1-mm membranes used to fill the post-enucleation cavity. L-PRF membranes were applied in a layered fashion: first as a stabilizing membrane over the BIOPAD barrier, then as a scaffold-fill material throughout the post-enucleation cavity (Figure 1G). This dual-layer technique provided both barrier protection and osteogenic stimulation. Following the hemostasis, the flap was sutured with 3.0 atraumatic silk (Doğsan, Trabzon, Türkiye). The patient was prescribed amoxicillin 1 g twice daily, dexketoprofen trometamol (NSAID) 2–3 times daily, and 0.12% chlorhexidine antimicrobial mouthwash postoperatively. A histopathologic examination of the material reveals the presence of non-keratinized stratified squamous epithelium, accompanied by chronic inflammatory cell infiltration and a sequential arch pattern within the epithelium (Figure 1H). In the postoperative control visits, intraoral healing was uneventful with satisfactory soft-tissue closure and long-term follow-up (12 months). Both clinical and radiographic examinations confirmed complete resolution of the lesion without recurrence (Figure 1I,J).

### 2.2. Case Two

**Figure 2:**
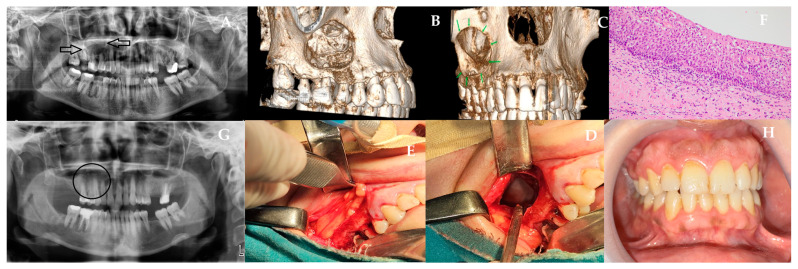
(**A**) Panoramic radiograph showing (black arrows) well-defined radiolucency extending posteriorly to tooth 17. (**B**,**C**) CBCT reconstructions showing sinus extension and thinning of the buccal cortical plate. (**D**) Intraoperative view of cyst enucleation. (**E**) Closure of vestibular perforation using buccal fat pad flap and placement of BIOPAD on the sinus membrane. (**F**) Histopathology demonstrating parakeratotic stratified epithelium with a palisaded basal layer. (**G**,**H**) 12-month postoperative clinical and radiographic images demonstrating stable healing and no sinus complications (black circle).

Technical and surgical challenges may arise during procedures involving the nasal cavity floor and maxillary sinus floor mucosa, particularly depending on the integrity of the bony borders between the nasal floor and the medial wall of the maxillary sinus. In this situation, a 45-year-old female patient presented to our clinic with swelling and tenderness in the right upper jaw. The maxillary sinus lesion was spreading towards the lateral aspect of the right nasal cavity floor. The patient’s medical history did not include any prior trauma. She had a daily smoking habit but no history of systemic disease. On initial presentation to our clinic, localized firm intraoral swelling was observed. When the patient was evaluated with panoramic film (Figure 2A) and CBCT (Figure 2B,C), a well-defined, unilocular, cortical radiolucency was observed extending from tooth number 15 to 17. The cyst demonstrated significant extension into the maxillary sinus region, creating potential for chronic sinusitis and fistula formation, a scenario requiring preventive soft tissue reconstruction. A preliminary diagnosis of RC was made based on these clinical findings. The surgery was performed under general anesthesia. A vestibular incision was made within the oral cavity, extending from tooth 13 to 16. The full-thickness mucoperiosteal flap was elevated. Subsequently, intraoral cyst enucleation was performed (Figure 2D). The vestibular perforation area of the cyst cavity was closed with bichat adipose tissue obtained by incision and blunt dissection distal to tooth number 17. The perforated thin nasal mucosa was closed with an absorbable sponge barrier (BIOPAD, Euroresearch, Italy) (Figure 2E). This combination of vascularized adipose tissue and absorbable barrier addressed both soft tissue bulk deficiency and epithelial separation, creating a dual-layer protective mechanism that prevents sinus complications. Extraction of tooth 17 with reduced bone support and tooth 16 with a focus of infection was performed. Following the attainment of hemostasis, the flap was closed using a 3.0 silk suture (Doğsan, Trabzon, Türkiye). The patient was prescribed Amoxicillin 1 g twice a day, NSAID Dexketoprofen Trometamol 2–3 times a day, and 0.12% chlorhexidine antimicrobial mouthwash postoperatively. A histopathologic examination of the material reveals a hyperchromatic basal cell layer consisting of cuboidal and columnar cells in parakeratotic multilayered epithelium with no rete ridge, showing palisification. The luminal surface exhibits parakeratotic epithelial cells that manifest a wavy appearance (Figure 2F). Postoperative control demonstrated stable soft-tissue healing with no signs of infection or sinus complications, and the 12-month follow-up confirmed complete clinical recovery and absence of recurrence (Figure 2G,H).

### 2.3. Case Three

**Figure 3:**
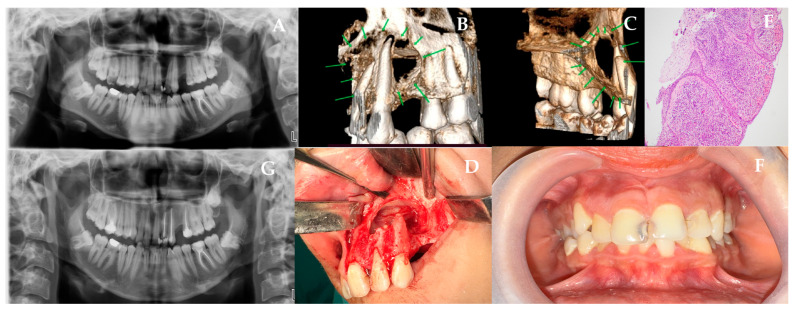
(**A**) Panoramic radiograph showing an unilocular lesion between teeth 21–26. (**B**,**C**) CBCT reconstructions confirming cortical thinning and nasal mucosa proximity (green arrows). (**D**) Intraoperative enucleation and apical resection of teeth 22–23. (**E**) Histopathology reveals non-keratinized squamous epithelium with chronic inflammatory infiltrate. (**F**,**G**) 12-month postoperative clinical and radiographic evaluation confirming resolution without recurrence.

A 17-year-old female presented to our clinic following the incidental detection of a radiolucent lesion during routine radiographic examination. Her medical history was unremarkable, with no prior trauma, systemic disease, smoking, or alcohol use. On clinical examination, there was no visible swelling. Panoramic (Figure 3A) and CBCT (Figure 3B,C) imaging revealed a well-defined, unilocular, corticated radiolucency extending from tooth 21 to 26. The area was firm on palpation without fluctuation. Vitality testing showed that teeth 21 and 23 were vital, while tooth 22 exhibited a delayed response. Based on these findings, a preliminary diagnosis of RC was made. The surgery was performed under general anesthesia. A full-thickness mucoperiosteal flap was elevated via an intraoral incision from the mesial of tooth 21 to the distal of tooth 26, with vertical releases. Upon identifying the bony perforation, the remaining thin cortical fragments were removed. Subsequently, intraoral cyst enucleation was performed (Figure 3D). Apical resection was performed on teeth 22 and 23 with retrograde obturation using Mineral Trioxide Aggregate (MTA, Angelus, Brazil), preserving anatomically important anterior maxillary teeth. For Case 3, L-PRF was prepared using the same standard protocol described above (2700 rpm, 12 min). Platelet-rich fibrin (PRF) was derived from the patient’s own blood and subsequently applied to the perforated nasal mucosal base. Following the hemostasis, the flap was closed using a 3.0 silk suture (Doğsan, Trabzon, Türkiye). The patient was prescribed Amoxicillin 1 g twice a day, NSAID Dexketoprofen Trometamol 2–3 times a day, and 0.12% chlorhexidine antimycobial mouthwash postoperatively. Histopathologic examination showed the presence of squamous epithelium that has not formed keratin, with several layers of cells. There is also chronic inflammation and a pattern of cells arranged in a sequence (Figure 3E). Early postoperative evaluation revealed smooth mucosal healing and stable closure of the surgical site, while the 12-month follow-up confirmed full restoration of the area with no residual pathology or recurrence (Figure 3F,G).

### 2.4. Case Four

**Figure 4:**
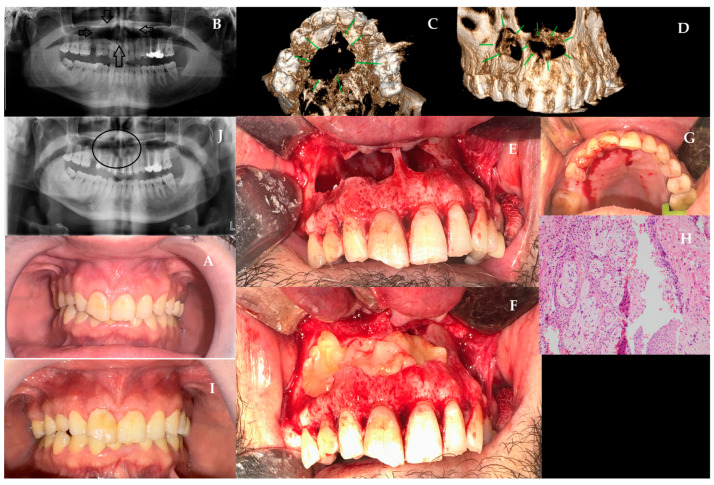
(**A**) Initial intraoral appearance showing vestibular fullness. (**B**) Panoramic film indicating a large radiolucency from tooth 14 to 23 (black arrows). (**C**,**D**) CBCT reconstructions showing nasal floor perforation and lesion expansion. (**E**) Intraoperative cyst enucleation with the extraction of an impacted canine. (**F**) Combined closure using PRF membranes and absorbable collagen sponge (BIOPAD). (**G**) Semi-rigid splinting of anterior teeth following apical resection. (**H**) Histopathology showing non-keratinized squamous epithelium with cholesterol clefts and chronic inflammation. (**I**,**J**) 12-month postoperative clinical and radiographic images showing full healing and no recurrence (black circle).

A 41-year-old male patient presented to our clinic with swelling in the maxillary region and requested the extraction of a canine tooth previously identified as impacted. The patient’s medical history indicated a childhood trauma to the anterior region; however, no radiographic examination had been performed for many years due to the absence of symptoms. A comparison of the panoramic radiograph taken two years before the current image revealed a substantial increase in the size of the lesion. The systemic history was unremarkable, with no tobacco or alcohol use. At the time of the patient’s initial visit to our clinic, a slightly firm, non-fluctuant swelling was detected in the vestibule of the maxillary incisors (Figure 4A). Electric pulp testing revealed that teeth 11, 12, 14, 15, and 23 were non-vital, whereas teeth 21 and 22 were vital. Panoramic film (Figure 4B) and CBCT (Figure 4C,D) showed a well-defined, unilocular, well-defined radiolucency with corticated borders extending from tooth number 14 to 23. This case presented the most complex scenario: a large cyst involving the entire anterior maxilla with multiple non-vital teeth, an impacted canine, and nasal floor perforation, requiring coordinated surgical, endodontic, and prosthodontic planning. CBCT sections of this lesion showed that it originated from the apices of the devital incisors and was unrelated to the enamel-cementum junction of the affected canine. Root canal treatments were performed on the infected teeth that primarily caused this condition. The surgery was performed under general anesthesia. A full-thickness mucoperiosteal flap was elevated through an intraoral vestibular incision. Following the observation of the perforation in the bone, the removal of the remaining thin cortical parts was conducted. Subsequently, intraoral cyst enucleation was performed (Figure 4E). The impacted canine tooth in the cyst cavity was then extracted. Apical resection was performed on teeth 12, 11, 21, and 22 with simultaneous retrograde MTA filling to stabilize the root apices. L-PRF was prepared according to the standard protocol described above. The perforated area was strategically closed with a combined approach: PRF membranes provided the regenerative scaffold while an absorbable sponge barrier (BIOPAD, Euroresearch, Italy) may help epithelial collapse at the nasal floor (Figure 4F). Teeth with root resection, for which mobility was predicted to increase with the weakening of bone support in the mouth, were splinted as semi-rigid (Figure 4G). Following the hemostasis, the flap was closed using a 3.0 silk suture (Doğsan, Trabzon, Türkiye). The patient was prescribed Amoxicillin 1 g twice a day, NSAID Dexketoprofen Trometamol 2–3 times a day, and 0.12% chlorhexidine antimycobial mouthwash postoperatively. A histopathologic examination of the material reveals the presence of non-keratinized multilayered squamous epithelium, accompanied by chronic inflammatory cell infiltration and a sequential arch pattern within the epithelium. Cholesterol clefts have also been observed in this area (Figure 4H). The initial postoperative visit showed uneventful soft-tissue healing despite the extent of the defect, and by 12 months, the region remained fully healed with no clinical or radiographic signs of recurrence (Figure 4J,I).

### 2.5. Case Five

**Figure 5:**
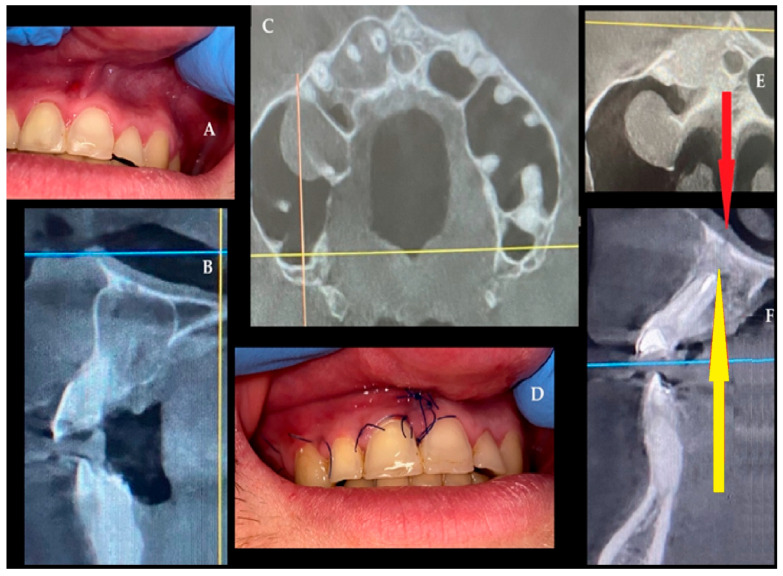
A situation when RC is causing visible cortical swelling and expansion with extended toward the right maxillary sinus alveolar recess without its perforation (**A**–**C**). A slight superior protrusion of the RC is noted towards the nasal floor, causing a small 1–2 mm bone dehiscence (**B**). RC Radicular cyst associated with maxillary incisors (11 and 12) extending toward the nasal floor without significant nasal mucosal involvement. (**B**) CBCT coronal section demonstrating superior extension of the lesion toward the nasal floor with focal thinning and minimal perforation, without elevation or adhesion of the nasal mucosa. (**C**) Axial CBCT slice illustrating the relationship between the cystic cavity and the roots of teeth 11, 12, and 13, with partial root protrusion into the lesion. (**D**) Postoperative view after enucleation and apicoectomy of teeth 11 and 12. (**E**,**F**) Graft placement showing filling of the defect (yellow and red arrows) with allogeneic bone mixed with platelet-rich plasma to create a “sticky bone” construct.

The presented case highlights how an RC was related only to just two maxillary incisor teeth, 11 and 12, during slow growth and progression, causing maxillary labial cortical bone swelling (A), asymmetry, and extended toward the nasal floor, causing a slight perforation of the nasal floor (Figure 5). The status of the lesion did not cause a significant loss of the nasal floor, and no adhesion with the nasal mucosa or elevation of the nasal floor was noted. A generally healthy 35-year-old male without any comorbidities was diagnosed because of painful maxillary swelling. Two teeth (11,12), which were necrotic, were treated endodontically with MTA (MTA, Maxi, Cerkamed, Stalowa Wola, Poland) along with tooth 13, whose root was partially embedded into the cyst cavity (C). After surgical preparation of the teeth, additional oral hygiene measures were performed, and teeth 22, 21, 11, 12, 13, and 14 were splinted using a composite splint (Splint, Arkona, Warsaw, Poland). Initial surgery took place under local anesthesia (LA) with 3 ampules (1.7 mL each) of Ubistesin Forte (articaine with epinephrine, 3M, Maplewood, MN, USA) because there were no signs of nasal cavity involvement. A standard mucoperiosteal flap was elevated using a VISTA approach, involving a split of the upper labial frenulum and extension into the gingival sulcus to create a full-thickness envelope flap. The entire cyst was enucleated, with surgical high-speed burs used to perform a localized ostectomy and partial apicoectomy of teeth 11 and 12 (yellow arrow). The entire wound cavity was filled with allogeneic bone graft (RCKiK, Katowice, Poland) that was mixed with patients’ blood and PRP to create a “sticky-bone” (E,F). No membranes, flaps, sponges, or other means had to be used because no cortical perforation to the palate or extrusion towards the nasal cavity was present. Bone material was condensed thoroughly throughout the entire cavity. Wound was sutured with 4-0 interrupted vertical mattress sutures (Dafilon, B Braun, Aesculap AG Am Aesculap-Platz, Tuttlingen Germany (D). Healing was uneventful and after three years no recurrence was observed. Special considerations include several factors. Adhesion of a radicular cyst (RC) or other cystic lesion to the palatal mucosa may require the use of a collagen membrane or a hemostatic dressing (BloodSTOP^®^, Life Science Plus, Mountain View, CA, USA) to stabilize the thin mucosa and reduce the risk of fistula formation. In addition, destruction of the anterior nasal spine (ANS) and involvement of the anterior nasal cavity or the nasopalatine canal require careful and effective local anesthesia. In selected cases, particularly when patient comfort and surgical access are concerns, general anesthesia may also be considered. Furthermore, when the labial cortical bone remains intact and the lesion perforates only the palate or the nasal floor, the lesion may be approached using either a palatal flap or a labial flap (horizontal incision approximately 5 mm above the mucogingival junction), allowing adequate access while avoiding incision within the anterior maxillary esthetic zone. Final histopathology concluded an occurrence of RC.

### 2.6. Case Six

**Figure 6:**
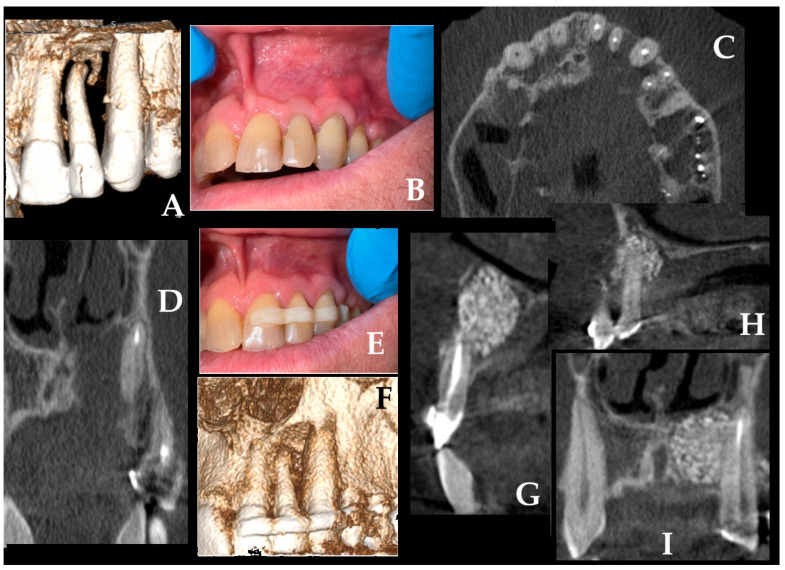
Another situation appears when the RC spreads within the nasal cavity floor, causing its destruction less than 1 cm, however, without spreading towards the maxillary sinus. Extensive anterior maxillary radicular cyst involving teeth 21–24 with destruction of the nasal floor and palatal cortical plate. (**A**,**B**) Preoperative CBCT images demonstrate a large cystic lesion encompassing four anterior teeth and extending superiorly toward the nasal cavity. (**C**,**D**) Axial and sagittal CBCT views showing root involvement and close approximation to the nasal floor. (**E**,**F**) Clinical photographs after endodontic treatment and buccal–palatal composite splinting of teeth 21–11–12–13–24 to stabilize pathologic mobility (tooth 22). (**G**) Intraoperative view after enucleation, ostectomy, and apicoectomy with placement of a xenograft–allograft “sticky bone” mixture within the defect. (**H**) Coverage of the nasal and palatal bony defects using a double-layer approach combined with a BloodSTOP and OsseoGuard collagen membrane. Grafted bone compressed on the two-layer solution with a good post-operative result. (**I**) Postoperative follow-up imaging at 2 years demonstrates satisfactory bone regeneration without recurrence or complications.

Another noteworthy scenario arises when a radicular cyst develops in the anterior maxilla and involves four anterior teeth (21–24) within its extent (Figure 6A,B). Four teeth (21–24) that were necrotic and/or embedded with the cyst lining were treated endodontically with MTA (MTA, Maxi, Cerkamed, Stalowa Wola, Poland) (A–D). A generally healthy 45-year-old male reported an atypical pain within the anterior nose. Improved diagnostics concluded the occurrence of a cystic mass. The lesion itself caused destruction of the nasal cavity floor and was spreading towards the maxillary sinus recess, however, without its destruction. After surgical preparation of the teeth, additional oral hygiene measures were performed, and teeth 21, 11, 12, 13, and 24 were splinted with a composite splint on both the buccal and palatal aspects (Splint, Arkona, Warsaw, Poland) (E,F). Before surgery, microbiological swab from nose revealed the presence of Staphylococcus aureus (S.A.), which required local intranasal application of Bactroban (Mupirocine, ointment 20 mg/g (2%); 15 g GlaxoSmithKline, London, UK) 3 times/day for 5 days. Second, a double-splint technique was applied because tooth 22 exhibited third-degree pathological mobility, and both the patient and surgical team sought to preserve the tooth and attempt to maintain it in situ without the need for extraction. A typical muco-periosteal flap was elevated with a Vista approach—split of the upper lip frenulum and then extended to the gingival sulcus as an envelope flap. Owing to the destruction of the lateral aspect of the anterior nasal spine and perforation of the nasal floor, surgery was performed under general anesthesia. With the Obwegeser Periosteal elevators (Obwegeser 38-630 06-07-38-630-11-07 17.5 cm/6 7/8”, KLS Martin, Tuttlingen, Germany), the muscles and mucosal membrane from the ANS and the anterior part of the nasal floor were lifted, ensuring that the nasal cavity bone floor is fully visible. The adhesion between the RC and the nasal mucosa was dissected bluntly with a scalpel blade. Bone ostectomy with apicoectomy of teeth 21–24 was made. Because the defect was quite big, a mixture of “sticky bone” composed of 1 g xenograft bone (XB) (1 g The Graft 0.25–1 mm, Manufacturer Purgo Biologics Inc., Seongnam-si, Republic of Korea) and 5 cm^3^ allogeneic bone graft (ABG) (RCKiK, Katowice, Poland) was placed within the defect. A resorbable collagen membrane (RCM) (15 × 20 mm-OsseoGuard Membrane-Zimmer Biomet, Collagen Matrix Inc., Oakland, CA, USA) was placed on the nasal bone deficit aspect and the palatal bone deficit along with a BloodSTOP hemostatic dressing (HD) was pressured along the bleeding naso-palatal canal (Life Science Plus, Mountain View, San Jose, CA, USA). Bone packing was sufficient and enabled good healing (G–I). A two-year follow-up revealed proper healing without any complications. Several important considerations arise in this context. When tooth extraction is required, the extent of the incision should be planned to allow adequate soft-tissue coverage of the extraction site and the grafted bone, thereby promoting proper socket healing. When a combined intranasal and intraoral approach is necessary, assessment of the nasal microflora is advisable to minimize the risk of bacterial spread, graft contamination, and subsequent inflammation or purulent discharge. Finally, when feasible, the VISTA approach through the upper labial frenulum may be preferred, as it provides adequate surgical access while maintaining favorable esthetic outcomes and minimizing visible scarring. Final histopathology concluded an occurrence of RC with inflamed lining.

### 2.7. Case Seven

**Figure 7:**
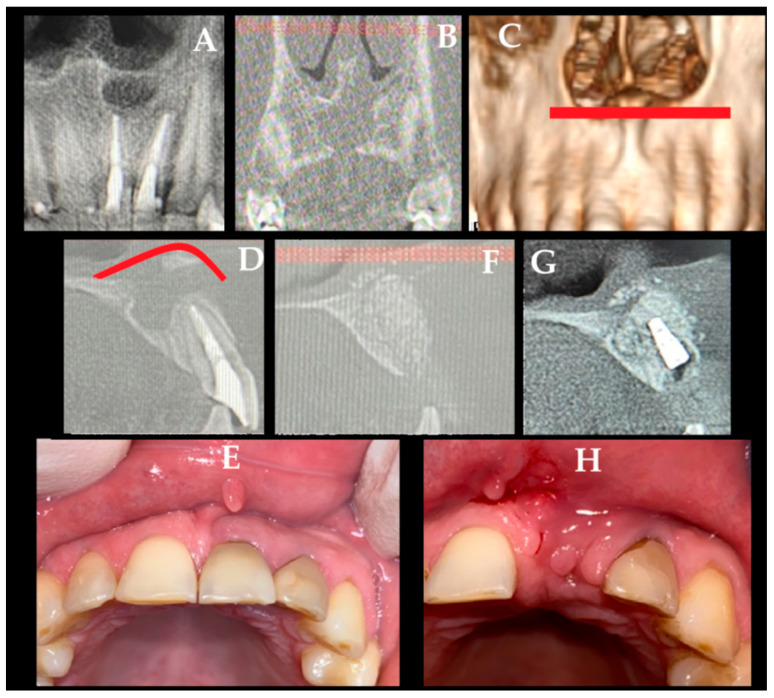
A residual cyst enlarged in size over time, resulting in the resorption of 21 teeth and apex fracture, and the necessity to remove the tooth with its RC and prepare for a dental implant placement in the second stage of surgery (**A**,**B**). Because of the scope of surgery related to a single tooth, but the lesion itself was advancing within the nasal floor and causing the elevation of the nasal mucosa, a need for a wider approach was necessary ((**C**,**D**)—red line marks the scope of subperiosteal elevation within the nasal floor). (**E**)—horizontal approach 5 mm above the mucogingival junction to provide better exposure to the nasal floor and later grant more reliable healing and approach towards the final procedure focused on a dental implant placement. Horizontal scar within the upper lip mucosa doesn’t affect future dental implant procedure, but on the other hand, it grants a very good exposure to the nasal floor. The result from bone grafting was good (**F**) and granted adequate dental implant placement (**G**). The scope of scaring and gingival projection was satisfactory (**H**), for more dental esthetics, a soft tissue gum transfer is considered.

When a combination of RC from teeth 21 is present with a recurrence of another cystic cavity, the scope of the bone approach is quite different. A residual cyst (DC, dentigerous cyst) after a previous surgery treated about seven years ago enlarged in size, shape, and protruded towards the nasal floor, causing some patient discomfort (Figure 7). A 50-year-old patient had a past surgery performed because of an atypical cyst in the anterior maxilla area within the naso-palatal duct; the histopathological type of the lesion is unknown. A resorption of 21 teeth and an apex fracture resulted in the necessity to remove these teeth and the cyst as well in its periapical area. The cyst relapse caused a significant loss of cortical buccal bone and spread of the cyst from under the left incisors (21,22, B–D) towards the palate and nasal floor. The destruction of the nasal floor was 12 mm in its longest diameter. Teeth 22, because of poor reaction to cold stimulus was treated endodontically with MTA (MTA, Maxi, Cerkamed, Stalowa Wola, Poland) (A,D). Nasal microbiological screening revealed no Staphylococcus aureus. After surgical preparation of the teeth, additional oral hygiene measures were performed. The first step of surgery was done under general anesthesia and with the use of horizonal approach 5 mm above the mucogingival (C,D, red line) junction to provide better exposure to the nasal floor and later grant more reliable healing and approach towards the final procedure focused on a dental implant placement (E). Horizontal scar within the upper lip mucosa doesn’t affect future dental implant procedure, but on the other hand, it grants a very good exposure to the nasal floor (E). After removal of the 21 incisor teeth with RC, local full vestibular and palatal muco-periosteal flaps were elevated, and bone curettage with ostectomy was performed via two approaches. Firstly, from the alveolar socket of the removed tooth, and a second approach from the ANS region, the lost cortical buccal bone, and the nasal cavity floor. Before the burr ostectomy, the nasal mucosa along with the anterior part of the nasal septum was elevated with Obwegeser elevators to grant access to the nasal septum, coagulate the area of Kisselbach plexus, keep a clear visibility to the nasal mucosa, and carefully bluntly divide the nasal floor mucosa from the cyst relapse. The horizontal incision grants a very good access to the nasal cavity and enables a more anatomical dissection within the anterior part of the nasal cavity. A mixture of XB and AGB, and the placement of two membranes, RCM + HD (E–H), supported the nasal mucosa and granted a good base for soft tissue support. From the superior part of the alveolar ridge, 21 teeth were closed with an RCM and sutured so that a secondary intention healing would be promoted. This was quite important for the future second stage of the approach. After six months under LA, a typical H-gingival incision was made to place a dental implant in the grafted and fully healed bone (G,H—Neodent, Straumann Group, Basel, Switzerland). The implant healed well; a three-year follow-up revealed stable bone position, no recurrence. A future gingival graft could be done to improve local anterior maxillary frontal area esthetics. This situation raises several important considerations. Adequate exposure of the anterior nasal base and anterior nasal spine is essential, and assessment of the nasal microbiological status may contribute to more favorable bone healing and reduced contamination of grafted material. The presence of any gingival fistula necessitates excision followed by closure with two healthy mucosal margins. A palatal approach should be considered when substantial palatal bone loss is present, and supraperiosteal elevation is preferred over submucosal dissection to promote more predictable healing outcomes. Final histopathology concluded a connective tissue mass with an RC.

### 2.8. Important Anterior Maxilla Surgical Remarks

Each surgical case in the anterior maxillary region should take into account the dental status and the potential presence of tooth-related inflammation or bacterial presence in the nasal cavity. When bone grafts or other biomaterials are used, possible bacterial contamination from the nasal or oral cavities, as well as compromised dental conditions, may adversely affect healing and lead to purulent complications. Conversely, following procedures involving the nasal mucosa, patients should be instructed to perform saline nasal irrigations and use xylometazoline nasal drops for five days postoperatively to support mucosal healing and reduce congestion. When nasal bleeding is present, a temporary 12/24 h anterior nasal packing should be made.

First, preoperative assessment of tooth vitality, endodontic status, mobility, and periodontal condition is essential because persistent apical infection or tooth instability may compromise graft integration and increase the risk of recurrence. When tooth preservation is intended, timely endodontic therapy with appropriate splinting should be considered to stabilize the dentoalveolar segment during healing.

Secondly, when the lesion approaches the maxillary sinus or nasal floor, careful evaluation of membrane integrity and mucosal status is required to prevent postoperative sinusitis, oroantral communication, or mucosal collapse into the defect. If mucosal compromise is suspected, separation and protection of the membrane with a barrier (collagen sponge/membrane) and adjunctive regenerative support should be planned. The extent of bone loss (<5 mm vs. >10 mm) requires different surgical strategies, and should guide whether a one-stage radical procedure with bone grafting, marsupialization, a two-stage decompression followed by definitive surgery, or alternative approaches are selected.

Thirdly, the incision design should prioritize adequate exposure of the anterior nasal base while preserving the esthetic zone; minimally visible approaches (VISTA via the upper labial frenulum) were preferred when feasible, as in Cases 5 and 6. In extensive defects or when intranasal dissection was anticipated, a wider horizontal incision 5 mm above the mucogingival junction was selected to provide safer access, improved visualization, and more controlled membrane handling (Case 7). Additional patient-related factors, such as the need for future dental implant placement, also influence the extent and design of the surgical incision.

Fourth, large cavities benefit from a combined regenerative strategy using xenograft or allograft mixed with PRP (‘sticky bone’) to improve handling and stability, together with collagen sponges or resorbable membranes to protect the nasal or sinus lining. In our series, this approach resulted in stable bone regeneration and uneventful long-term healing (2–3 years in Cases 5–7), without fistula formation or recurrence. Most cases in this series fell within Groups B–D (Table 1), reflecting lesions with compromised bony boundaries or actual communication with the nasal cavity floor and/or maxillary sinus floor that required adjunctive regenerative measures, mucosal protection, or staged surgical management.

## 3. Discussion

Cysts are defined as pathological cavities lined with epithelium and filled with fluid or semi-fluid, with the potential for growth. These lesions are broadly categorized as odontogenic, arising from tooth-associated tissues, or non-odontogenic, originating from non-dental structures. Lesions can be found in both the jawbone and the soft tissue [30]. Inflammatory cysts of the jaw are a group of odontogenic lesions. The etiology of this condition is attributed to epithelial remnants in the periodontal ligament, arising from apical periodontitis subsequent to the loss of vitality and necrosis of the pulp [31]. The diagnosis of RC is typically made during a routine radiographic examination or in the aftermath of an acute exacerbation [32]. Some lesions were detected incidentally during routine radiographic examination, whereas others presented with swelling, tenderness, pain, or functional concern. In a recent and exhaustive study by Du et al., it was reported that RCs generally manifest asymptomatically; however, when they attain a substantial size, they can be diagnosed with symptoms such as swelling and pain [11]. RCs grow slowly and can lead to root resorption and tooth displacement [31]. Teeth can be preserved when they are either in a stable position within the bone or have undergone endodontic treatment and splinting to maintain their condition. When fractures with pathological resorption are present or the teeth are fully embedded within the cystic lesion, their prognosis is poor. Teeth with a relatively favorable crown-to-root ratio were retained after apical resection and retrograde filling. The indication for the extraction of cyst-related teeth was based on the following criteria: severe loss of material, a crown-to-root ratio below 1/1, and the presence of bone resorption in the approximal region. In case 4, the anterior group, which had an inadequate crown/root ratio after resection, was splinted as a semi-rigid splint to absorb the destructive bite forces. The aim of this approach was to avoid crestal resorption of the vestibular cortical bone and extensive bone loss, while maintaining tooth continuity in the anterior esthetic region. Otherwise, no displacement or root resorption was observed in our cases. Radiographically, RCs are usually circular in shape with cortical and distinct borders at the apical part of the nonvital tooth. The peripheral regions of the RC exhibit hyperostotic borders that persist along the lamina dura. In infected and rapidly growing cysts, these limits may not be observed [31]. Occasionally, the coexistence of two or more cystic lesions or other pathologies substantially alters the surgical approach and significantly influences the extent, size, and configuration of the resulting bone defect. Our cases, in which patients presented asymptomatic, fit this general radiographic description and showed a uniform hyperostotic border. It was observed that only Case 4 exhibited an impacted canine tooth accompanied by radiolucency. The absence of an association between the cyst epithelium and the enamel cementum border of the impacted canine tooth, in addition to its origin from the apical aspect of the nonvital incisors, indicated a preliminary diagnosis of RC. Histopathology confirmed the diagnosis. The clinical and radiographic manifestations of numerous odontogenic cysts and tumors can bear a resemblance to those of an RC. Therefore, although clinical history, aspiration, and three-dimensional imaging are helpful in the differential diagnosis, histopathology is the gold standard. In the anterior maxilla, differential diagnosis is particularly critical because developmental cysts (e.g., nasopalatine duct cysts), odontogenic tumors such as ameloblastoma, and non-odontogenic lesions may closely mimic inflammatory cysts radiographically and clinically, especially when nasal floor elevation or palatal expansion is present. Therefore, lesions in this region require a cautious diagnostic algorithm integrating vitality testing, aspiration, CBCT assessment of sinonasal involvement, and mandatory histopathologic confirmation before definitive reconstruction. In extensive cases, further imaging studies are instrumental in monitoring the complete extent of the lesion and associated structures [33,34].

The cases, situated in the maxilla, were in proximity to the floor of the nasal cavity, the nasal mucosa, the maxillary sinus wall, and the sinus membrane. In such cases, the objective is to prevent anatomical cavities from communicating with the cyst cavity following cyst enucleation and to promote healing of the resulting defect. Although preservation of separate sinonasal and cystic compartments was favored in our stratification to minimize contamination and promote stable healing, we acknowledge that in rare salvage scenarios such as severely atrophic edentulous jaws with massive defects and non-reconstructable shared walls, intentional unification of the cyst cavity and maxillary sinus may be considered, provided that meticulous sinus drainage, mucosal management, and postoperative surveillance are ensured. As evidenced by the extant literature, the utilization of collagen membranes/pads, gelatin-based hemostatic sponges, and platelet-rich blood products has been documented for this purpose [35,36]. Recent evidence supports the effectiveness of L-PRF blocks and A-PRF + grafts in large bone defects following cystic lesion removal [37,38]. This approach aims to prevent chronic infections and the potential formation of fistulas resulting from the translocation of microbial flora. Additionally, it aims to maintain the homeostasis of the maxillary sinus and nasal cavity. Recent research has deepened the understanding of surgical outcomes in odontogenic cyst management. Enucleation has been shown to achieve bone regeneration comparable to graft-assisted techniques, with no significant difference in volumetric healing at 12 months, suggesting that the regenerative capacity of the maxillary bone and periosteum can often suffice when the cyst is fully removed and the defect stabilized [39]. Evidence also indicates that the addition of platelet-derived biomaterials, such as PRP or PRF, can enhance bone shrinkage and accelerate soft-tissue healing compared with enucleation alone or marsupialization [40,41]. While marsupialization remains a conservative option for large cysts near vital structures, it frequently necessitates secondary enucleation; approximately three-quarters of initially marsupialized cases ultimately require definitive surgery [42,43]. Collectively, these findings emphasize that surgical planning should consider cyst size, anatomical proximity, and the likelihood of epithelial persistence, with timely definitive enucleation supported by biologically active scaffolds offering outcomes comparable to more complex reconstructive procedures. Nonetheless, further prospective trials are warranted to quantify the long-term regenerative benefits of adjunctive biomaterials across different cyst types and anatomical sites. From a clinical standpoint, treatment of cystic lesions in the anterior maxilla can vary from conservative endodontic therapy or decompression to complete surgical enucleation, with or without adjunctive regenerative procedures. The choice of approach depends on factors such as lesion size, proximity to the nasal cavity or maxillary sinus, tooth prognosis, and the risk of residual epithelial tissue. In our series, cases with nasal or sinus involvement were preferentially managed with definitive enucleation combined with biologically active scaffolds, allowing a single-stage procedure while limiting postoperative morbidity. Simon described two types of RCs [44]. The first form is a true RC containing a continuous cavity completely lined with epithelium. The other form is a periapical cyst, also known as a pocket/bay cyst. The epithelium is attached to the edges of the apical foramen, and the cystic lumen is open to the affected root canal. Nair’s analysis revealed that 61% of these cysts were confirmed as true cysts, while the remaining 39% were classified as pocket cysts [45]. Histopathologically, RCs are completely or partially covered with squamous epithelium. The lumen of the cyst contains fluid with a low protein concentration and a collection of multinucleated giant cells and cholesterol clefts. The intensity of these contents may vary in acute and chronic inflammation [46]. The accumulation of cholesterol crystals is the result of the breakdown of lymphocytes, macrophages, red blood cells, and plasma cells. In the present study, histopathologic analysis revealed the presence of non-keratinized multilayered squamous epithelium accompanied by chronic inflammation, sequential arches, cholesterol clefts, and a hyperchromatic basal layer of cuboidal and columnar cells in parakeratosis. Although rare, squamous cell carcinoma has been reported to develop from the epithelium of RCs [47,48]. Some authors suggest that incomplete enucleation may raise the risk of epithelial metaplasia, though this remains controversial [49]. In our case series, all lesions representing true cysts were completely enucleated. The disadvantage of this procedure is the risk of damage to the surrounding anatomical structures and neighboring teeth while enucleating large cysts. Particularly in cases 1 and 2, perforation of the nasal mucosa and sinus membrane caused surgical difficulties. The iatrogenic relationship was minimized by the use of PRF and an absorbable sponge barrier. In their study, Zhao et al. [50] employed PRF in the treatment of bone cavities associated with RCs. They posited that the fibrin present in PRF would serve as a clot matrix, with the potential to stimulate angiogenesis through the action of growth factors [50]. In the second case, the bichat fat was repositioned and fixed under the flap with absorbable sutures. This procedure was performed to prevent future fistulas in the vestibule of the cavity and to increase soft tissue thickness. The buccal fat pad flap was used in our series to obliterate cystic cavities adjacent to the maxillary sinus wall, providing a well-vascularized and low-morbidity option for defect closure. Kim et al. similarly reported comparable outcomes, demonstrating that buccal fat pad mobilization results in predictable soft-tissue healing and minimal donor-site complications in oral and maxillofacial reconstruction [51]. In the third case, the perforated buccal area and nasal mucosa were both closed with PRF. In the fourth case, the perforated area was closed with PRF and absorbable sponge barrier (BIOPAD). Surgical access to anterior maxillary lesions should be individualized: most cases in this series were treated via intraoral vestibular or VISTA approaches to preserve facial esthetics, while wider horizontal incisions and combined intranasal–intraoral dissections were chosen in the presence of anterior nasal spine destruction or firm mucosal adhesion. When necessary, a supplementary intranasal approach was used. A variety of methodologies were employed and subsequently followed up in seven distinct cases of RCs in the maxilla. In all instances, the cyst cavity was associated with the nasal mucosa or the maxillary sinus floor, necessitating the specific regenerative and protective protocols described. We have followed our patients for at least a period of one year postoperatively with no evidence of recurrence, and follow-up evaluations are ongoing. Nevertheless, although inflammatory cysts like RCs typically carry a low recurrence rate following complete enucleation, recent data indicate that recurrence, while uncommon, may still occur several years after surgery, underlining the need for long-term surveillance [11,52]. In conclusion, in the treatment of RCs, early diagnosis, appropriate endodontic and surgical intervention, and the use of adjunctive methods that support tissue regeneration were associated with favorable clinical outcomes in this series. However, further comparative studies are needed to determine if these approaches significantly reduce complications or accelerate healing compared to traditional techniques. All patients included in our case series achieved complete recovery with no evidence of sequelae; follow-up evaluations are ongoing. The necessity for prospective studies employing larger samples is evident in order to adequately evaluate the long-term outcomes of the various treatment approaches utilized in the management of RCs. It is essential to evaluate the status of the maxillary sinuses (e.g., polyps, mucosal thickening, retention cysts, reduced patency of the osteomeatal complex, and fluid accumulation), as well as the nasal cavity (e.g., fluid or purulent secretions, elevation of the nasal floor, septal deviation, and nasal discharge), in order to determine whether additional intranasal or endoscopic procedures (FESS/ESS) are required to maintain adequate sinus drainage and a secretion-free local environment [27,28,53,54,55].

In the first four cases (Cases 1–4), the defects were considered suitable for spontaneous bone healing due to the presence of relatively stable residual bony walls; therefore, biologically supportive approaches such as PRF and collagen pads were preferred. In contrast, in cases presenting with larger bone defects and requiring additional structural support (Cases 5–7), bone grafting procedures were performed to promote bone regeneration. This approach was particularly preferred in the anterior maxillary region to preserve or reconstruct adequate bone volume for future prosthetic rehabilitation and potential implant placement, considering the esthetic and functional demands of this region.

Study limitations include a small number of patients with the presence of typical RC arising from the anterior maxilla towards the nasal cavity floor with various scopes of nasal bone loss, propagation towards the maxillary sinuses, and the necessity of simultaneously performing teeth removal or the use of other technical means.

## 4. Conclusions

In conclusion, the management of radicular cysts spreading towards the sinonasal cavities benefit from a structured, anatomy-based approach. Our preliminary observations suggest that combining enucleation with regenerative materials may support stable clinical outcomes and maintain anatomical integrity. However, as this is a descriptive case series, these results should be interpreted with caution. All patients included in our case series achieved complete recovery with no evidence of sequelae; follow-up evaluations are ongoing. The necessity for prospective studies employing larger samples is evident in order to adequately evaluate the long-term outcomes of the various treatment approaches utilized in the management of cysts.

## Figures and Tables

**Table 1:** Scope of similarities and differences in surgical approaches to the nasal cavity floor (NCF) and maxillary sinus floor (MSF).

Approach/Location	Nasal Cavity Floor (NCF)	Maxillary Sinus Floor (MSF)
A. Lesion contained within bone borders	No communication to NCF expected.	No communication to MSF expected.
B. Lesion adjacent to cavity (thin bone, “at-risk”)	No communication initially; communication may occur after enucleation Prophylactic collagen barrier advised.	No communication initially; communication may occur after enucleation Prophylactic collagen barrier advised.
C. Lesion extends toward cavity with dehiscence/perforation (single-compartment communication)	Communication present/likely Elevate the nasal mucosa and perform two-layer separation (mucosa + barrier/PRF).	Communication is present when the Schneiderian membrane is perforated Membrane protection/repair (PRF + collagen pad/membrane).
D. Lesion extends toward NCF and toward MSF (shared wall loss/dual-risk)	Bony wall destruction increases risk of creating a common defect Consider staged surgery (decompression, definitive enucleation) when stability/closure is uncertain.	Avoid converting the defect into a “single cavity” connecting the sinus and cyst space Maintain separation with robust compartmental closure or use a staged strategy.
E. Lesion occupies both NCF and MSF (combined cavity involvement)	One-step radical surgery if closure is reliable, or two-step decompression/marsupialization when a large defect/contamination risk exists.	One-step radical surgery if closure is reliable, or two-step decompression/marsupialization when a large defect/contamination risk exists.

**Table 2:** Summary of clinical features, surgical interventions, and outcomes for the presented cases.

Case	Age/Sex	Primary Symptom	Anatomic Group	Surgical Approach & Regenerative Strategy	Histopathological Diagnosis	Follow-Up and Outcome
1	33 M	Asymptomatic	B/C	Enucleation; BIOPAD (collagen sponge) + L-PRF	Radicular Cyst (Residual)	12 months; complete healing, no recurrence
2	45 F	Swelling/Pain	C	Enucleation; Buccal Fat Pad (BFP) flap + BIOPAD	Radicular Cyst	12 months; stable healing, no sinus complications or recurrence
3	17 F	Asymptomatic	B	Enucleation; Apicoectomy (#22, 23) + MTA + L-PRF	Radicular Cyst	12 months; full resolution, no recurrence
4	41 M	Swelling	C/D	Enucleation; Impacted canine extraction; Apicoectomy (#11, 12, 21, 22) + MTA + Splinting; BIOPAD + L-PRF	Radicular Cyst	12 months; full healing, no recurrence
5	35 M	Painful Swelling	B	Enucleation; Apicoectomy (#11, 12); Splinting; Allogeneic bone + PRP (“sticky bone”)	Radicular Cyst	3 years; no relapse
6	45 M	Atypical Nasal Pain	D	Enucleation; Apicoectomy (#21–24); Splinting; Xenograft/Allograft + PRF (“sticky bone”); OsseoGuard + BloodSTOP	Radicular Cyst	2 years; satisfactory bone regeneration, no recurrence or complications
7	50 F	Discomfort	D/E	Recurrent lesion; Extraction (#21); Grafting (XB/AGB) + RCM/HD; Delayed Dental Implant	Radicular Cyst	3 years; stable bone, no recurrence, implant stable

Abbreviations: M: Male; F: Female, BFP: Buccal Fat Pad, MTA: Mineral Trioxide Aggregate, PRF: Platelet-Rich Fibrin, L-PRF: Leukocyte and Platelet-Rich Fibrin, PRP: Platelet-Rich Plasma, XB: Xenograft Bone, AGB: Allogeneic Bone, RCM: Resorbable Collagen Membrane, HD: Hemostatic Dressing, #: tooth number.

## Data Availability

The data presented in this study are available on request from the corresponding authors due to patient privacy and ethical restrictions.
